# Early Childhood Development Is Not Enough: In Defense of Children with Developmental Delays and Disabilities and Their Right to Family-Centered Early Childhood Intervention (In the Global South)

**DOI:** 10.3390/children11050606

**Published:** 2024-05-18

**Authors:** Claudine Störbeck

**Affiliations:** The Wits Centre for Deaf Studies, School of Education, Faculty of Humanities, University of the Witwatersrand, Johannesburg 2000, South Africa; claudine.storbeck@wits.ac.za

**Keywords:** early childhood intervention, early childhood development, children with disabilities, family centered intervention

## Abstract

The international recognition of the critical importance of the early childhood phase has been firmly established through decades of rigorous research, evidence-based practices, and undeniable evidence of the returns on investment made during this formative period. Consequently, early childhood development has emerged as a top priority on both national and international agendas. This momentum reached a pinnacle in 2015 with the unanimous adoption of the 17 Sustainable Development Goals (SDGs) by the United Nations, which placed a particular emphasis on children under the age of five within the education-focused SDG 4, notably target 4.2, centered on ensuring that all girls and boys are ready for primary education through the provision of accessible “quality early childhood development, care and pre-primary education”. However, the Global South reflects the glaring omission of addressing the needs of children at risk of poor development due to disabilities. This paper underscores the imperative for specialized early childhood intervention tailored to young children with disabilities and their families, commencing as early as possible following birth. It advocates for Early Childhood Intervention (ECI) as a service distinct from general Early Childhood Development (ECD), emphasizing the crucial role of families as active partners from the outset. Furthermore, the paper strengthens the case for Family-Centered Early Childhood Intervention (Fc-ECI) through the integration of evidence-based practices and an in-depth description of one such program in South Africa with specific reference to deaf and hard-of-hearing infants and their families. This model will be guided by core concepts outlined in WHO and UNICEF Early Childhood Intervention frameworks. Through this exploration, the paper aims to shed light on the urgent need for inclusive approaches to early childhood development, particularly for children with disabilities, and to advocate for the adoption of Family-Centered Early Childhood Intervention as a cornerstone of global efforts to ensure the holistic well-being and development of all children.

## 1. Introduction

In November 2023, the global population reached 8 billion, revealing a demographic landscape where more than 26% of the population was below the age of 15 years old and 650 million (8.1%) were within the earliest phase of birth to five years old. Over 40% of this burgeoning early childhood population are from the Global South [1,2]. Associated with the surge in population, the world has made substantial progress on infant survival rates, with infant mortality rates dropping by almost 60% from 1990 to 2020 [3,4]. Reduced mortality rates in pre-term and NICU babies have, in turn, contributed to the growth in “at-risk” infants, with an increase in morbidities such as developmental delays and disabilities [5,6,7]. This population of children with disabilities below the age of five years is estimated to be 49.8 million, and Sub-Saharan Africa and South Asia account for more than half of the children with disabilities [8,9]. Identified as “the most vulnerable population” within the global health and early childhood landscape [10], children with developmental disabilities remain largely invisible on the global stage [11,12] with little progress towards prioritizing their needs in global early childhood development initiatives [13,14]. The next section will briefly define the field of Early Childhood followed by descriptions of the key concepts, including early childhood development, early childhood intervention, and, finally, family-centered early childhood intervention.

## 2. Defining Early Childhood

Early Childhood is often categorized as the birth to eight-year-olds cohort of children, with Early Childhood Development (ECD) defined as the attainment of “the cognitive, physical, language, motor, social and emotional” milestones during that time [15]. Recently there has been a rise in the prioritization of the first 1000 days—conception to two years of age—with its heightened sensitive periods and brain plasticity [16,17]. Additionally, the research and early childhood community have consistently included the “next 1000 days” of the two- to five-year-olds as just as critical [18]. In this paper, early childhood will be classified as the first five years of life, the period of life in which 90% of the child’s brain develops [10]. Shonkoff [19] argues that the “brain science story” makes for undeniable evidence for the case for investment in these critical early years.

As we begin to explore the implementation of intervention and support in the early years, it is important to note that there are various terms that are used interchangeably when referring to the field: Early Childhood Development (ECD), Early Childhood Education (ECE), Early Childhood Education and Care (ECEC), Early Childhood Education and Development (ECED) and Early Childhood Care and Education (ECCE) [20]. In this article, the term Early Childhood Development will be used, as encompassing the breadth of what is meant by these interchangeable titles, unless otherwise specified.

## 3. Early Childhood Development

The early 2000s saw the international community’s growing awareness of the critical early infant period, catalyzed in part by the “unprecedented convergence of child development and research” [19,21]. This groundswell of global interest was further strengthened with the results of long-term research showing the negative impact of early childhood risks such as poverty-related maternal health and environmental issues, including inadequate nutrition, unhygienic living conditions, and exposure to infections and local pollutants and toxins without the necessary access to healthcare [22,23]. Additional risk factors to typical development were identified as parental stress and mental well-being, neglect, exposure to violence, safety, the lack of secure housing, as well as insufficient responsive caregiving and stimulation, stunted growth and delayed early childhood development.

Longitudinal studies revealed that high-risk experiences during early childhood inevitably led to delayed and stunted early childhood development as well as more long-term harmful effects on adult health [24], including chronic disease, lower educational attainment and earning power, as well as depression [25]. Low- and Middle-Income Countries (LMIC), representing 43% of children under the age of five, were identified as the most vulnerable communities of the world with the highest risk of their young children not reaching their full potential [26]. To mitigate these developmental delays, James Heckman [18], Nobel Laureate and Professor of Economics, advocated for the economic benefits of investing in early childhood, demonstrating that the earliest investments yield the most significant returns. While examining these returns on investment, Heckman’s research [19] revealed that, unlike the 7–10% returns on investing in preschool programs for three- and four-year-olds, investment in high-quality birth-to-five early childhood development programs yielded a 13% return on investment.

With these robust foundations firmly established, early childhood was elevated to the forefront of the global health agenda of key organizations such as the United Nations (UN), United Nations Children’s Fund (UNICEF), United Nations Educational, Scientific and Cultural Organization (UNESCO), the World Health Organization (WHO), and the World Bank, all committed to the imperative of leaving no child behind (The UNICEF Mandate and fundamental SDG Principle). This culminated in the integration of early childhood into the Sustainable Development Goals (SDG 4) during the 2015 United Nations Summit. Target 4.2 of the SDGs mandates that by 2030 “all girls and boys have access to quality early childhood development, care, and pre-primary education so that they are ready for primary education” [27]. This historic inclusion meant that for the first-time governments would be accountable for demonstrating progress in achieving Indicator 4.2.1, which measures the “proportion of children under five years of age who are developmentally on track in health, learning and psychosocial well-being.” Currently however, it only includes children from two to five years of age using UNICEF’s Early Childhood Development Index (ECDI2030) [28]. With the recent addition of a new measure, the WHO’s Global Scales for Early Development (GSED) [29], it is anticipated that this indicator will soon return to its original wording, thereby enabling the inclusion of all children from soon after birth to five years of age.

To this end, the Nurturing Care Framework (NCF) was developed as a global initiative for ECD as a pathway with five core areas: health, nutrition, responsive caregiving, security and safety, and early learning [4]. Although presented as a framework for all children in ECD services, initially the NCF was mainly conceptualized to include children under five who were at risk of developmental delays due to stunting and poverty, thereby excluding “children with disabilities” [30]. For this reason, general ECD programs, policies, and frameworks—such as the NCF among others—are “vastly inadequate to address the most pressing needs of children with developmental disabilities” [4]

## 4. Disability in Early Childhood

Traditional models of disability have predominantly adhered to the medical model, which defines disability as a physiological impairment resulting in a medical condition. However, in 2001 the World Health Organization (WHO) introduced the International Classification of Functioning, Disability and Health Framework (ICF) to establish a standardized framework and terminology. This initiative aimed to promote a more uniform description and understanding of disability over the life course [31].

The ICF framework transcended the limitations of the medical model by embracing the biopsychosocial approach, incorporating functionality and context into existing medical definitions. This expanded framework underscored the distinction between childhood disability and adult disability, leading to the development of the International Classification of Functioning, Disability and Health Framework for Children and Youth [32]. Informed by the enriched ICF framework, this paper defines childhood disabilities as “long-term physical, mental, intellectual, or sensory impairments present from the earliest years, affecting and hindering child development, functional capabilities, and participation in their environments and society on an equal basis with others” [31,32,33]. 

In line with understanding this childhood disability, the global burden of disease estimates suggest that nearly 53 million children under the age of five have developmental disabilities [5] encompassing conditions such as epilepsy, intellectual disability, autism spectrum disorder, and attention deficit/hyperactivity disorder. Alarmingly, 95% of these young children are reported to reside in low- and middle-income countries (LMICs) [10].

Compared to children without disabilities, the UN [32] reported that children with disabilities are 34% more likely to experience developmental stunting, 24% less likely to receive responsive care and early stimulation, and 25% less likely to attend early childhood education. Long-term impacts further underscore these disparities: children with disabilities are 42% less likely to possess foundational reading and numeracy skills, 49% more likely to have never attended school, and 20% less likely to harbor expectations of a better life [8,32,34]. 

The prevalence of childhood disabilities and their significant impact on typical infant and early childhood development, coupled with the “higher risk of being exposed to abuse and neglect, stigma, and discrimination” pp. 438-44 [4], underscore the urgent need for specialized intervention. Support and intervention for these children with disabilities have thus far been noticeably neglected, highlighting the imperative for both prioritization and urgency on the global agenda, in additional to the current focus typical for early childhood development.

However, despite the global surge in support and the corresponding increase in funding for early childhood development by 121% between 2007 and 2016 [35], funding for disability declined by −11.4%. Olusanya and her colleagues reported in 2022 that they had not found any evidence of funding increases since their initial report [4].

The imperatives are clear: (i) Early Childhood Development needs to become inclusive (IECD), ensuring that children can remain in their local communities and access their equal rights to the significant investments in ECD alongside their peers, (ii) ECD environments need to be made accessible and inclusive to all children, and (iii) ECD practitioners must be equipped with the necessary skills to understand and address the learning needs of the most vulnerable children within the ECD sector, particularly children with disabilities. To achieve these goals, newborn screening needs to be prioritised where indicated, as well as early and regular developmental screenings be implemented to ensure early identification and diagnosis of potential developmental delays and disabilities. This should always be followed by a prioritized support system and rapid to referral toto ECI services comprising home-based Family Centered Early Childhood Intervention (Fc-ECI) as evidenced in ECI best practice.

## 5. Early Childhood Intervention

The field of Early Childhood Intervention (ECI) was established to provide immediate specialized support and intervention aimed at facilitating the optimal development of children with disabilities. In their foundational ECI Handbook, Meisels and Shonkoff [36] described ECI as services that “promote child health and well-being, enhance emerging competencies, minimize developmental delays, remediate existing or emerging disabilities, prevent functional deterioration and promote adaptive parenting and overall family function” (p xvii). Because of the complexity of the needs and, therefore, the support that children with disabilities need, successful ECI necessitates multidisciplinary teams willing to work in an integrated and transdisciplinary manner [36,37,38]. Limbrick [39] refers to the individual medical professionals providing this multidisciplinary input and support as the “team around the child” (TAC). This multidisciplinary team may include the following professionals among others:

Medical Doctors and Specialists: Pediatrician, Otolaryngologist, Ophthalmologist, Neurologist, Orthopedic surgeon, Psychiatrist;
Healthcare Professionals: Social Worker, Psychologist, Optometrist, Dentist;
Allied Health Professionals: Occupational Therapist, Physiotherapists, Speech and Language Pathologist, Nutritionist;
Early Childhood Professionals: Early Childhood Developmentalist, Early Interventionist, Pediatric Carer, Pre-school Teacher;
Educators: Specialist Teachers (Autism/Deaf/Blind/Neuro Diverse, etc.);
Partners and Stakeholders: Parent Support Partner, Community Role Model (e.g., Deaf Adult, Blind Adult, Autistic Adult), Family Support Group.

Professionals in early childhood must undergo a conceptual shift from viewing a child in isolation, focusing solely on their disability and interventions offered in isolation, to recognizing themselves as part of a team supporting a child with disabilities within an intricate, interconnected, socio-ecological context [36,39,40,41,42]. In addition to the traditional team of health professionals, ECI requires a primary support specialist called an Early Interventionist or Early Intervention provider, who is equipped to support the unique needs of each young child during their earliest years and offers the ECI support within the home environment on a frequent and consistent basis.

Whilst many professionals remain integral to a child’s long-term developmental journey—for example, a child with cerebral palsy may continually require the expertise of a physiotherapist and nutritionist, as well as regular visits to primary medical and healthcare professionals such as their physician and orthopedic surgeon—Early Childhood Interventionists should strive to empower families toward independence. This comprehensive ECI support entails providing families with the information, knowledge, and skills necessary to maximize their unique child’s optimal development—cognitively, linguistically, physically, and socio-emotionally—whilst honoring and respecting each family’s diverse culture, needs, and aspirations [37,43,44].

While Early Childhood Intervention (ECI) programs typically span three to five years or until the child transitions to formal schooling, their impact should be lasting and continue to be relevant going forward. ECI should equip parents for the ongoing journey ahead by imparting them with the necessary problem-solving skills and the ability to access information as it evolves. Moreover, it should foster strong parent–child relationships and instill in parents the confidence to trust their instincts.

To establish a sustainable foundation for parents of children with disabilities, a traditional expert-driven, child-centered early intervention process is insufficient. Instead, the aim should be a longer-term, parent-guided, reciprocal relationship characterized by mutual respect and co-creation of meaning [45]. What sets apart this form of Early Childhood Intervention (ECI) for children with disabilities from all other forms of Early Childhood Education/Development (ECE/ECD) is the fundamental role of parents in what we term “Family-Centered Early Childhood Intervention” (F-ECI).

## 6. Family-Centered Early Childhood Intervention

Hippocrates, often regarded as the father of Western medicine, is reported to have said that “it is more important to know what sort of person has a disease, than to know what sort of disease a person has”, and yet it took almost two and a half millennia for person-centered general medical care to become a priority in practice [46]. When working in pediatrics, however, person- and family-centered approaches had already begun to gain momentum in the 1950s and 1960s through the pioneering work of Gesell, Kirk, and Brazelton [47,48,49,50]. 

These pioneers made a profound impact on professionals in early childhood education and development who work with children with disabilities, as they encountered the limitations of medical–therapeutic models. The field of Early Childhood Intervention, particularly in sensory disabilities, such as vision and hearing loss, emerged as a leader in innovative, family-centered approaches to practice. One noteworthy example is the SKI-HI early intervention program for families with deaf and hard-of-hearing children (birth to three) launched in 1971 [51,52,53]. The underlying philosophy and key features of the SKI-HI early family-centered intervention model, often regarded as a benchmark for best practices, include the following:

(i) Commencement of intervention as close to birth as possible to leverage optimal brain plasticity during the early months and years of life;
(ii) Adoption of a family-centered approach that is based on collaborative partnership with the family, recognizing them as the most important and consistent element in the infant’s life;
(iii) Recognition that early intervention is most effective when conducted in the natural environment (home) of the child and family, utilizing daily routines and activities as contexts for learning;
(iv) Provision of unbiased information tailored to the family’s goals, enabling them to make informed choices for their child’s development;
(v) Emphasis on communication and language at the core of the early intervention program, fostering trusting and responsive socio-emotional relationships within the family while laying foundations for the child’s cognitive development [54].

Each Fc-ECI home visit with the family should prioritize collaboratively developed short-term and end goals, guiding small, frequent steps over the course of three to five years. For the child, this entails fostering happiness and optimal development tailored to their unique abilities across various domains, while cultivating a profound sense of love and acceptance. Concurrently, the family should be sufficiently equipped and empowered with agency and self-efficacy to parent effectively, take the lead in decision-making, and advocate for their child, all while maintaining a sense of well-being and optimism [55,56,57,58].

The above components of Fc-ECI are all deeply grounded in the strengths-based paradigm [59,60], which means that the early interventionist looks at what the young child can do and the strengths he or she brings, as opposed pathologizing these young children and focusing on their disability and what they cannot do. In the same way, the strengths and indigenous knowledge and skills of the family are considered assets in the intervention process whether the family members are educated and literate or not. The field of Fc-ECI acknowledges the privilege of home-based visits, and interventionists require mature and advanced personal insight and reflection to ensure that they are able to respect and unconditionally accept and value all families. This goes beyond mere “cultural competence” to practicing early intervention cultural reciprocity and humility [44], whether it is a couple, an un-wed teenage mother, a multigenerational family living in one small venue, a child-headed household, or a family unit from a culture and religion foreign to one’s own.

One of the fundamental goals of Family-Centered Early Childhood Intervention (Fc-ECI), which differentiates it from medical and therapeutic interventions, is for the early interventionist to work themselves out of a job toward the families’ becoming autonomous and confident, internally as they engage with their child at home, as well as externally as they advocate for their child’s unique needs and rights on their journey ahead.

An example of one such national Family-Centered Early Childhood Intervention programe is the HI HOPES program of South Africa. The components of will now be practically explored from the perspective of an evidence-based Fc-ECI program in South Africa. Although the program being shared supports families and their deaf and hard-of-hearing children, the guiding principles of Fc-ECI remain constant within the spectrum of childhood disability and family support. The foundational building blocks and principles of Fc-ECI programs are as follows: (i) Early and continuous, (ii) Individualized, (iii) Comprehensive and focused on holistic development, (iv) Intensive and coordinated, (v) Transdisciplinary/interdisciplinary and team-based, (vi) Evidence-informed and outcomes-driven, (viii) Unbiased and family-led partnerships, (ix) Home-based and focused on daily life and natural routines, and (x) Accountable and quality assured [38,43,51,53,61,62].

## 7. Evidence-Based Practice in a Family-Centered Early Childhood Intervention Program for Families and Their Children with Deafness in a LMIC: A South African Case Study 

Despite the international prominence that early childhood development has attained, the urgency to meet the early needs of young children with disabilities has been largely neglected for most of these children and their families living in LMICs. Where there has been active progress, the focus has been on medical and therapeutic interventions. In South Africa, this has been particularly evident within early childhood and hearing loss, where, even though amplification options are multiple and therapeutic and medical expertise is high, Fc-ECI had never been an option before HI HOPES.

To this end, in 2006 the first home-based Family-Centered Early Childhood Intervention program, HI HOPES, was launched in South Africa to support families and their deaf and hard-of-hearing young children from birth to three and, where needed, up to six years of age. Prior to the launch, we worked strategically within this highly politicized and polarized field, known for the longstanding “great debate” between the auditory–oral–medical and the visual–manual–social paradigms. We, therefore, engaged with multiple stakeholders in the field to ensure that there was a shared understanding of the neutrality of the Fc-ECI principles of HI HOPES amongst the stakeholders, including the Deaf Community, Audiologists, Speech and Language Therapists, Otolaryngologists, as well as Educators and ECD practitioners. In addition to creating a shared understanding, we also assured stakeholders that the aim would be one of teamwork and interdisciplinarity.

Our international research led us to the SKI HI model of Fc-ECI, with its philosophy of parent partnerships through parent-led education and support through an un-biased process to equip all parents to make their own informed choices for their unique child. The intentionality and depth of the three-year curriculum provided intensive resources that had been researched and evidence-based. The continuous and consistent home-visits focused on holistic development of deaf and hard-of-hearing children within the comfort of daily life and routines, as well as prioritizing the importance of individualized support. Over the extensive period of HI HOPES support, the model included the fundamental principle of quality assurance and accountability through the mentoring of early interventionists and the consistent monitoring of the child’s developmental progress [43,51,52,53,62]. 

The expanse of the two-volume, 2167-page curriculum provided evidence of the two core principles and content we were seeking: (i) the equipping and empowerment of the parents for the breadth of information and skills they would require on their journey, and (ii) the holistic and optimal development of the unique child in preparation for the early childhood education (ECE) system. We knew that, in terms of the inequalities in our South African history, we would need to find a way to ensure consistency of quality and content for all infants and families no matter their racial or cultural profile [44,63,64], geographic region, or socio-economic status. Additionally, we wanted to ensure that, despite being within the Global South, we would work toward international best practice in ECI, including the top training and the top curriculum along with rigorous mentoring and quality assurance. The final goal of responsible transitioning out of HI HOPES once families felt confident and autonomous showed us that this was indeed the model and program we wanted to embrace. A core ingredient in our success was the invaluable support and commitment from the established SKI HI Institute and their expert trainers and mentors as we began the first Fc-ECI program in South Africa. After almost twenty years, HI HOPES remains the only Fc-ECI program in the 16 member states of the Southern African Development Community (SADC), to the extent possible to date. Since launching in 2006, HI HOPES has been invited to share programmatic experiences, data, training, and advice for other LMICs wanting to start Fc-ECI: Austria, Australia, England, Greece, Iran, Italy, Kazakhstan, Korea, Morocco, Moscow, Namibia, Russia, Scotland, Senegal, Spain, and the USA.

As a multi-cultural country with 12 official languages—often referred to as the “rainbow nation”—we made a concerted effort to ensure that wherever possible HI HOPES families could communicate in their home language during the home-visits. Early interventionists of all ethnicities and cultures were trained in the regions of service so that we could ensure authentic, culturally responsive support from some locals “just-like-them”. This has been one of the biggest successes of HI HOPES and subsequently led to receiving permission to translate our SKI HI resource-handouts for families into local South African languages. Home-visits always included both parents wherever possible, making sure that fathers always felt included. The visits needed to be fully active and participatory and encouraged participation by the whole family including siblings and grandparents and whoever was visiting at the time. Adult learning principles were used, to ensure that the process was respectful to the adult family members, guided by their questions and areas of concern and interest. In addition to information and knowledge, the interventionists would often model skills whilst facilitating a natural process of learning and upskilling by family members. Weekly (<12 months) and bi-weekly (>13 months) home visits usually lasted about an hour and were typically activity-based with a growth in complexity over an extended period of time. The array of goals was developed in partnership with the family and were monitored and adjusted where necessary on a four-monthly basis. The aim was that each child developed optimally in all domains of child development, with a core focus on age-appropriate expressive and receptive language and communication skills, within the typically bilingual or multilingual family contexts

Monitoring and Evaluation became a central part of the HI HOPES program from the start, as we wanted to ensure (i) our families and children received the highest quality of appropriate and effective intervention as possible, (ii) the interventionists received the necessary guidance and mentoring, and (iii) progress and outcomes were recorded and monitored for both administrative and quality assurance reasons. In its 18th year of existence, the HI HOPES data has become the largest longitudinal dataset on disability in Africa [43,51,52,65]. In addition to the home interventionist (HI), all HI HOPES families were invited to join the Deaf Mentor (DM) program, which was developed to introduce families to deaf adults, whatever the language or amplification choices, as role models of success and the achievements that deaf people in South Africa can achieve. This included introductions to Deaf Culture and deaf ways of being, as well as the education, language, and communication choices that were available. If parents wanted to learn South African Sign Language as part of a bi/multi-lingual choice, Deaf Mentors could offer that as well. Finally, HIs always ensured that HI HOPES families had opportunities to meet other families with deaf or hard-of-hearing children through social events and personal introductions, and very often these relationships would blossom into long-term family friendships.

As HI HOPES has grown from one to currently six of the nine provinces, as well as extending our support to also include visual and other multiple disabilities, we have been encouraged by the “in-principle” support and goodwill from local and national government. Despite this support, as well as South Africa’s public commitment to the UN and UNICEF imperatives for the rights of early childhood and children with developmental disabilities, no significant funding has yet been made available. The Center for Deaf Studies at the University of the Witwatersrand in Johannesburg, South Africa, committed to fundraise for HI HOPES due to the deep belief that families and their deaf or hard-of-hearing infants and young children (who often also had additional challenges) had the right to Fc-ECI support beyond the very well established medical and therapeutic infrastructure of South Africa. With almost 20 years of successfully implementing the HI HOPES Fc-ECI program in the country with growing goodwill and support from both the private and public sectors—Health, Education, and Social Development among others—the country has still not “put its money where its mouth is”.

In line with the rights of children with disabilities [33,66], mirrored in the ECI trends internationally, HI HOPES has remained free at the point of need [65]. Though beneficial, this community engagement and long-term financial support from civil society for services that government should be funding cannot be seen as a long-term solution. Despite this, the HI HOPES Fc-ECI program has grown into over 70% of the country and has developed the largest longitudinal dataset of its kind, thereby ensuring that the positive outcomes and successes of the program move beyond qualitative, community-centered anecdotes to evidence-based practice using national (population-level) data.

In summary, the HI HOPES Fc-ECI model advocates for a comprehensive, multidisciplinary approach to partnering with and supporting families and their infants and toddlers who are deaf or hard-of-hearing, with the central aim of informing and equipping parents to make their own informed decisions based on the particular needs of their infant without any bias in terms of language and communication approaches employed or type(s) of amplification used [52]. This, along with the socio-emotional support that families receive on their journey, has led to HI HOPES families learning to accept and embrace their deaf or hard-of-hearing child and finding ways to celebrate his or her unique strengths and abilities as opposed to laying blame at the feet of a parent or family member. We have seen this generation of deaf and hard-of-hearing children grow up with strong emotional bonds and relationships with their parents and siblings, something that had not been easy without the Fc-ECI. Data are ample and research is on-going. We hope to keep publishing our findings and results, whilst never losing sight of our hands-on work with families.

## 8. Conclusions

The critical importance of early childhood development and intervention, particularly for children with disabilities, in addressing the needs of a significant and often overlooked segment of the global population is unequivocal. The demographic landscape, with over 650 million children aged birth to five years old, underscores the urgency of prioritizing early childhood initiatives. While strides have been made in improving infant survival rates, the growing population of “at-risk” infants, particularly those with developmental delays and disabilities, necessitates tailored interventions to support their optimal development. The shift from traditional medical models to a biopsychosocial approach, as exemplified by the International Classification of Functioning, Disability and Health Framework, has broadened our understanding of childhood disabilities. However, despite advancements in early childhood development, children with disabilities continue to face significant barriers to accessing appropriate support and intervention, particularly in low- and middle-income countries (LMICs).

Family-Centered Early Childhood Intervention (Fc-ECI) emerges as a pivotal approach in addressing the unique needs of children with disabilities and their families. By prioritizing collaboration, empowerment, and individualized support, Fc-ECI aims to foster optimal development while equipping parents with the necessary skills and confidence to advocate for their child’s rights and well-being. The case study of the HI HOPES program in South Africa serves as a compelling example of the transformative impact of Fc-ECI in empowering families and promoting inclusive practices; however, the disparity in funding for disability-focused programs highlights the need for greater investment and prioritization of inclusive early childhood development initiatives.

Moving forward, there is a clear imperative for national governments and stakeholders within the fields of early childhood disability to prioritize investment into inclusive early childhood development initiatives. Comprehensive investment will need to include sufficient finances and resources, as well as expert capacity and time. Additionally, a parallel focus on strategic and culturally competent implementation will be required, along with public commitments and accountability. In their Global report on children with developmental disabilities [13], the WHO and UNICEF make responsibility clear:

“governments have the responsibility to respect and fulfil the human rights of all citizens, including children with developmental disabilities” who “should enjoy all human rights and fundamental freedom on an equal basis with other children” including “equality in opportunities to grow up in nurturing environments and to access early learning and education … to develop to the fullest … to grow up in a family environment; and to participate fully in family, cultural and social life, also recognizing the importance of family assistance and support”.

(pp. 29–30)

As professionals dedicated to early childhood development, we bear the responsibility of fostering environments where every one of our children, irrespective of their abilities, can flourish and achieve their utmost potential during these formative years of life. By adopting holistic, evidence-based methodologies to support families and their young children with disabilities, grounded in an unwavering commitment to holding our governments accountable, we can collectively ensure the realization of this vision.

## Data Availability

The data presented in published articles. The database is currently embargoed.
